# Medical financial hardship in the Southern United States: the struggle continues across generations pre- and post- the Affordable Care Act

**DOI:** 10.1007/s43999-024-00049-7

**Published:** 2024-09-04

**Authors:** Biplab Kumar Datta, Steven S. Coughlin, Justin Xavier Moore, Jie Chen

**Affiliations:** 1 Institute of Public and Preventive Health, Augusta University, Augusta, GA USA; 2 Department of Health Management, Economics and Policy, Augusta University, Augusta, GA USA; 3 Department of Biostatistics, Data Science and Epidemiology, Augusta University, Augusta, GA USA; 4 Department of Behavioral Science, University of Kentucky College of Medicine, Lexington, KY USA

**Keywords:** Financial toxicity, Regional disparity, Medical bill, Socioeconomic disparities, Generations, US South

## Abstract

**Introduction:**

Medical financial hardship in the United States is a growing public health concern. This study aims to assess the south vs. non-south disparities in medical financial hardship among US adults of different generations – Boomers (born between 1946 and 1964), Generation X (born between 1965 and 1980), and the Millennials (born between 1981 and 1996) across periods pre- and post- Affordable Care Act (ACA).

**Methods:**

This observational study utilizes data from multiple waves of the National Health Interview Survey (NHIS) split into three periods: pre–ACA (2011–2013), ii) post ACA (2015–2018), and iii) COVID-19 pandemic (2021–2022). Multivariable logistic regressions were fitted, separately for each generation in each period, to compare the extent of medical financial hardship among those from South to rest of the US, and Karlson-Holm-Breen (KHB) decomposition was applied to analyze whether there was a mediating impact of health insurance coverage.

**Results:**

Adults living in the South were more likely to experience medical financial hardship in all three periods. Residing in the South was associated with 1.7 to 2.6% points (pp) higher probability of medical financial hardship among boomers, 1.8 to 4.0 pp among generation Xers, and 1.7 to 2.8 pp among millennials. The relationship was robust after accounting for chronic comorbidities, sociodemographic and socioeconomic attributes and was partially mediated through differences in health insurance coverage.

**Conclusions:**

The problem of medical financial hardship has been deeply rooted in the South across generations, which was partly attributable to the regional differences in health insurance coverage.

**Supplementary Information:**

The online version contains supplementary material available at 10.1007/s43999-024-00049-7.

## Introduction

Geographic disparities in health outcomes in the United States (US), especially South vs. non-South differences, have been a known concern. Several studies showed higher prevalence of cardiovascular diseases and higher and diverging mortality trends in Southern states [1–3]. Part of the southern US has been considered as the “diabetes belt”, where prevalence of diagnosed diabetes was notably higher than rest of the nation [4]. South vs. non-South disparities in other health outcomes such as cardiometabolic conditions, self-reported health, cancer incidence and mortality, maternal mortality, and low birth weight were also documented [5–9]. The current study intends to expand the evidence base by exploring south vs. non-south disparities in medical financial hardship, which can simultaneously be a cause and consequence of other health disparities.

There is a mounting concern that the escalating costs of medical care are posing a significant financial burden for patients suffering from cancer, cardiovascular disease, and various other diseases [10–17]. Due to the rising health care prices along with lack of health insurance coverage and increased cost sharing, many patients in the US experience high out-of-pocket costs [17], ensuing a national medial debt burden of USD 220 billion [18].

The high costs of health care may lead to financial hardship and have negative effects on the well-being of patients and their families [12, 15, 16]. A recent study observed that cancer survivors living with medical financial hardship (defined as problems affording care or delaying or forgoing any care because of cost in the past 12 months) had up to a 17% increased risk of mortality when compared to cancer survivors without medical financial hardship [12]. Those who are low-income or uninsured face the greatest burden of financial hardships related to healthcare [14]. Patients who are affected by financial hardship are less likely to adhere to their prescription medications [15]. Financial distress is also associated with increased worry, depression, and anxiety [13, 15]. Using data from a nationally representative 10% panel of consumer credit reports between January 2009 and June 2020, a study estimated that the average medical debt in the U.S. was $429, with an estimated 17.8% of individuals having medical debt in 2020 [17].

To make health care more affordable and thereby to alleviate the financial strain from health care expenses, the Affordable Care Act (ACA) was enacted in 2010 and implemented in 2014 [19]. Since its implementation, the ACA has been associated with several positive health outcomes, such as increased rates of insurance coverage, improved access to care, and decreased healthcare-related bankruptcies. However, the ACA’s impact on health outcomes has varied across different states and was mostly studied in the context of Medicaid expansion [20, 21]. Few studies examined implications of the ACA on medical debt and financial burden [22–25], however, these studies did not offer much on the regional variations in medical financial hardship. Especially, less is known about whether and how geographic variations in healthcare-related financial hardship have evolved during pre- and post- ACA periods, and during the COVID-19 pandemic. Our knowledge is further limited about the experience of healthcare-related financial hardship by different generations, namely the Boomers (born between 1946 and 1964), the Generation X (born between 1965 and 1980), and the Millennials (born between 1981 and 1996), let alone the experience at the nexus of regional and generational differences.

This study aimed to address this gap by assessing regional differences in medical financial hardship within generations using a nationally representative sample of U.S. from 2011 to 2022. Of note, we did not intend to assess the impact of ACA on financial hardship, rather we explored the regional variation in financial hardship for the boomers, generation X, and millennials across different ACA timelines. Of particular interest was the occurrence of financial hardship in the South, where household-level access to credit and banking services is the lowest in the US [26]. Also, 8 of the 11 states that have not adopted or implemented Medicaid expansion till 2022, are in the South [27]. As such, assessment of medical financial hardship in the South by generation, compared to rest of the US may aid policy discussions and stakeholder engagements to mitigate healthcare-related financial distress.

The primary objective of this study, therefore, was to determine whether geographic region (US South versus all other regions) was associated with medical financial hardship, and whether there were noticeable differences before and after implementation of the ACA for the boomers, generation X, and millennials. The secondary objective was to assess whether the regional differences in medical financial hardship, within the generations, in pre- and post- ACA periods were attributable to health insurance coverage.

## Methods

### Data

In this observational study, we conducted a temporal analysis using pooled cross-sectional data on 186,689 adults (aged 18 + years), born between 1946 and 1996, from multiple waves of the National Health Interview Survey (NHIS) in the past 12 years. We performed analyses by splitting the survey waves into three periods as follows: (i) pre-ACA implementation period – 2011 to 2013, (ii) post ACA implementation – 2015 to 2018, and (iii) COVID-19 pandemic – 2021 to 2022. Survey waves were pooled into respective periods, in accordance with the NHIS guidelines for pooling survey data.^28^

The NHIS is a nationally representative surveillance system administered by the National Center for Health Statistics (NCHS). The NHIS annually collects self-reported data on various health topics via face-to-face interviews of civilian noninstitutionalized population of the US. The NHIS uses a multistage stratified sampling design to produce generalizable estimations and interviews are conducted in all 50 states and the District of Columbia [28]. We used publicly available anonymized secondary data from the Sample Adult files of the NHIS. The data used in this analysis met the definition of the NIH exempt human subject research (Exemption 4), and, therefore, ethics committee approval was not required.

The NHIS reports respondent’s age in years. A respondent’s year of birth was calculated by subtracting age from the survey year. Based on estimated birth years, respondents were grouped as boomers, generation X, and millennials. In 2014, when the ACA was implemented, boomers were aged 50 to 68 years, generation X were aged 34 to 49 years, and millennials were aged 18 to 33 years.

### Measures

Participants were defined as having any financial hardship from medical bills if they reported having problems paying or were unable to pay medical bills (including bills for doctors, dentists, hospitals, therapists, medication, equipment, nursing home or home care) in the past 12 months. Our outcome variables thus, was a binary variable indicating whether an individual had experienced financial hardship or not.

Our primary explanatory variable was a categorical variable comprising the four U.S. Census Bureau regions – Northeast (reference category), Midwest, South, and West. Our secondary explanatory was a binary variable indicating whether a respondent was from the South or from rest of the US (i.e., Northeast, Midwest, or West).

Our mediating variable, health insurance coverage, was a binary variable, indicating whether a respondent had any health insurance coverage (including private health insurance, Medicare, Medicaid, State Children’s Health Insurance Program (CHIP), state sponsored health plan, other government programs, or military health plan) or not.

### Statistical analysis

We first examined how proportion of individuals experiencing financial hardship and health insurance coverage among the boomers, generation X, and millennials differed across U.S. regions – South, Northeast, Midwest, and West in three study-periods. We performed adjusted Wald tests to assess whether the differences between the South and the other regions were statistically significant for respective generations and periods.

Next, to obtain odds of experiencing financial hardship for individuals living in the South, we estimated the following multivariable logistic regression models:

1 $$\:logit\left({FH}_{i}\right)={\beta\:}_{0}+\sum\:_{j=2}^{4}{\beta\:}_{1,j}Regio{n}_{j,i}+{\varvec{X}}_{\varvec{i}}{\varvec{\beta\:}}_{2}$$

Where, *FH*_*i*_ indicates whether individual *i* experienced financial hardship. The key explanatory variables, *Region*_*j, i*_, indicate whether individual *i* resided in *j*^*th*^ region (*j* ∈ [2, 3, 4]) or not. ***X*** is the vector of demographic and socioeconomic covariates including age, age squared, sex, race and ethnicity, educational attainment, family income (as share of Federal Poverty Line threshold), number of chronic comorbidities, and survey year fixed effects. Except age and age squared, all other variables included in vector ***X*** were categorical variables and categories of each variable are listed in Table 1.

**Table 1:** Descriptive statistics of the study population by generation and periods

	Boomers			Generation X			Millennials		
	2011-13	2015-18	2021-22	2011-13	2015-18	2021-22	2011-13	2015-18	2021-22
**Categorical covariates**									
**Region**									
Northeast	18.98	19.13	18.45	16.87	17.58	17.3	16.06	16.62	16.32
West	22.2	22.12	21.4	22.9	21.52	20.27	23.85	22.25	20.67
Midwest	36.82	36.61	37.86	36.3	36.5	39.12	35.95	35.92	37.36
South	21.99	22.14	22.28	23.93	24.39	23.31	24.14	25.22	25.65
**Sex**									
Male	48.8	48.18	47.6	49.35	48.75	49.12	49.73	49.85	49.61
Female	51.2	51.82	52.4	50.65	51.25	50.87	50.27	50.15	50.39
**Race**									
White	73.22	72.22	71.79	62.02	60.76	60.5	59.74	57.59	55.87
Black	11.26	11	10.64	12.44	12.06	11.91	13.56	13.89	12.81
Asian	4.36	4.79	4.94	6.4	7.5	6.4	5.54	6.64	7.16
Hispanic	10.39	10.95	10.83	18.32	18.39	18.82	20.1	20.63	20.77
Other	0.76	1.05	1.8	0.82	1.3	2.37	1.06	1.25	3.39
**Education**									
< HS graduate	12.05	11.32	10.58	12.62	11.33	10.56	13.49	8.12	7.02
HS diploma	26.31	25.51	28.28	22.96	22.02	24.8	25.68	21.91	24.92
Some college	30.37	29.95	28.2	29.91	27.93	26.14	37.97	33.92	26.48
College graduate	31.26	33.22	32.94	34.51	38.71	38.5	22.85	36.04	41.59
**Income**									
< 100% of FPL	10.61	8.89	8.52	13.25	9.82	8.55	22.4	14.24	10.32
100 to < 200% of FPL	13.68	14.81	16.81	17.39	15.68	14.37	22.09	18.97	18.22
200 to < 400% of FPL	27.01	25.69	28.68	30.1	26.69	27.06	28.99	30.72	29.18
≥ 400% of FPL	48.7	50.61	45.99	39.26	47.81	50.02	26.52	36.07	42.28
**Comorbidity**									
0	33.08	24.31	22.16	60.83	52.08	47.66	74.46	70.14	68.44
1	30.26	29.54	28.59	26.05	28.68	30.4	20.7	23.51	24.21
2	20.27	23.27	24.9	9.06	12.16	14.03	3.95	4.9	5.55
3+	16.39	22.88	24.35	4.05	7.08	7.9	0.9	1.46	1.80
**Continuous covariate**									
Age	56.32	39.51	24.55	61.28	44.47	28.52	65.65	48.97	32.95
Obs.	28,964	26,635	18,895	25,366	19,222	13,353	21,057	19,322	13,875

Note: For categorical covariates, percentages are reported. Percentages add to 100 across rows for respective characteristics. For continuous variables, means are reported. All estimates were obtained using complex survey weights. White, Black, Asian, and other refer to non-Hispanic White, non-Hispanic Black, non-Hispanic Asian, and non-Hispanic other, respectively. Chronic comorbidities include heart disease (coronary heart disease, angina pectoris, or myocardial infarction), stroke, hypertension, diabetes, cancer, arthritis, COPD, and asthma

Average marginal effects (AMEs) of residence in the South on financial hardship were estimated and pairwise comparisons of margins between the South and other regions (i.e., Northeast, Midwest, and West) were assessed. Bonferroni adjustments were made to adjust for the likelihood of increase in Type-I error.

Next, health insurance coverage was added to the model to examine whether the relationship between financial hardship and region was channeled through health insurance:

2 $$\:logit\left({FH}_{i}\right)={\alpha\:}_{0}+\sum\:_{j=2}^{4}{\alpha\:}_{1,j}Regio{n}_{j,i}+{\varvec{X}}_{\varvec{i}}{\varvec{\alpha\:}}_{2}+{\alpha\:}_{3}Insuranc{e}_{i}$$

Where, *Insurance*_*i*_ indicates whether individual *i* had health insurance coverage or not. A statistically significant estimate of *α*_*3*_ and |*α*_*1,j*_| < |*β*_*1,j*_| would be indicative of the relationship between financial hardship and region being mediated through health insurance coverage.

Next, to assess the likelihood of financial hardship among individuals living in the South with their counterparts living in rest of the US, we estimated the following models:

3 $$\:logit\left({FH}_{i}\right)={\gamma\:}_{0}+{\gamma\:}_{1}\:Sout{h}_{i}+{\varvec{X}}_{\varvec{i}}{\varvec{\gamma\:}}_{2}$$

4 $$\:logit\left({FH}_{i}\right)={\lambda\:}_{0}+{\lambda\:}_{1}\:Sout{h}_{i}+{\varvec{X}}_{\varvec{i}}{\varvec{\lambda\:}}_{2}+{\lambda\:}_{3}Insuranc{e}_{i}$$

Where, *South*_*i*_ is a binary variable that takes the value 1 if individual *i* resided in the South and 0 if in rest of the US (i.e., Northeast, Midwest, or West). Equations 1, 2, 3, and 4 were separately estimated for generations (i.e., boomers, generation X, and millennials) and study-periods (i.e., 2011-13, 2015-18, and 2021-22) for each generation. Since age is an important determinant of health insurance access (e.g., Medicare enrollment at age 65 years), as well as a natural risk factor for various chronic conditions that may influence medical financial hardship, AMEs (from Eq. 3) at different ages were estimated and reported.

Next, we separately interacted sex (male or female), race/ethnicity (non-Hispanic White, non-Hispanic Black, non-Hispanic Asian, Hispanic, or non-Hispanic other), educational attainment (less than high school graduate, high school diploma, some college, or college graduate), family income (< 100% of FPL, 100 to < 200% of FPL, 200 to < 400% of FPL, or ≥ 400% of FPL), and number of chronic comorbidities (0, 1, 2, or 3+) with *South*_*i*_ and estimated similar models stated in Eqs. (3) and (4). These specifications provide further information about the risk of financial hardship for Southerners of certain characteristics. For example, likelihood of experiencing financial hardship for males living in the South, females living in the South, non-Hispanic Whites living in the South, and so on. These exercises essentially served as sensitivity analyses of our original estimates and these results are presented as supplemental materials.

Lastly, to formally evaluate the mediating association of health insurance coverage, we applied Karlson-Holm-Breen (KHB) decomposition to decompose the relationship between financial hardship and South in direct and indirect (i.e., via health insurance coverage) components [29]. The direct part refers to the portion of the relationship net of health insurance coverage. The indirect part refers to the portion that is mediated through health insurance coverage. We also estimated the percentage (referred to as confounding percentage) by which the relationship between financial hardship and South was mediated by health insurance coverage. All other covariates, including chronic comorbidities, were considered as concomitant variables.

All analyses were conducted with Stata 18.0 software. Except for the KHB decomposition that does not support complex survey weights, all regressions were estimated using the NHIS sampling weights to account for the complex sampling design. Standard errors for the KHB decomposition analysis were obtained via bootstrapping with 100 replications. The level of significance was set at 5% level.

## Results

Our sample comprised 74,494 boomers, 57,941 generation Xers, and 54,254 millennials. Demographic and socioeconomic characteristics of adults by generation and study period (i.e., 2011-13, 2015-18, and 2021-22) are presented in Table 1. Distribution of generations within regions was fairly similar across the periods. While around 70% of the boomers were non-Hispanic White, the share gradually declined among generation Xers and the millennials. On the other hand, around 20% of the millennials were Hispanic, which was nearly 10% points (pp) higher than that among the boomers.

**Fig. 1:**
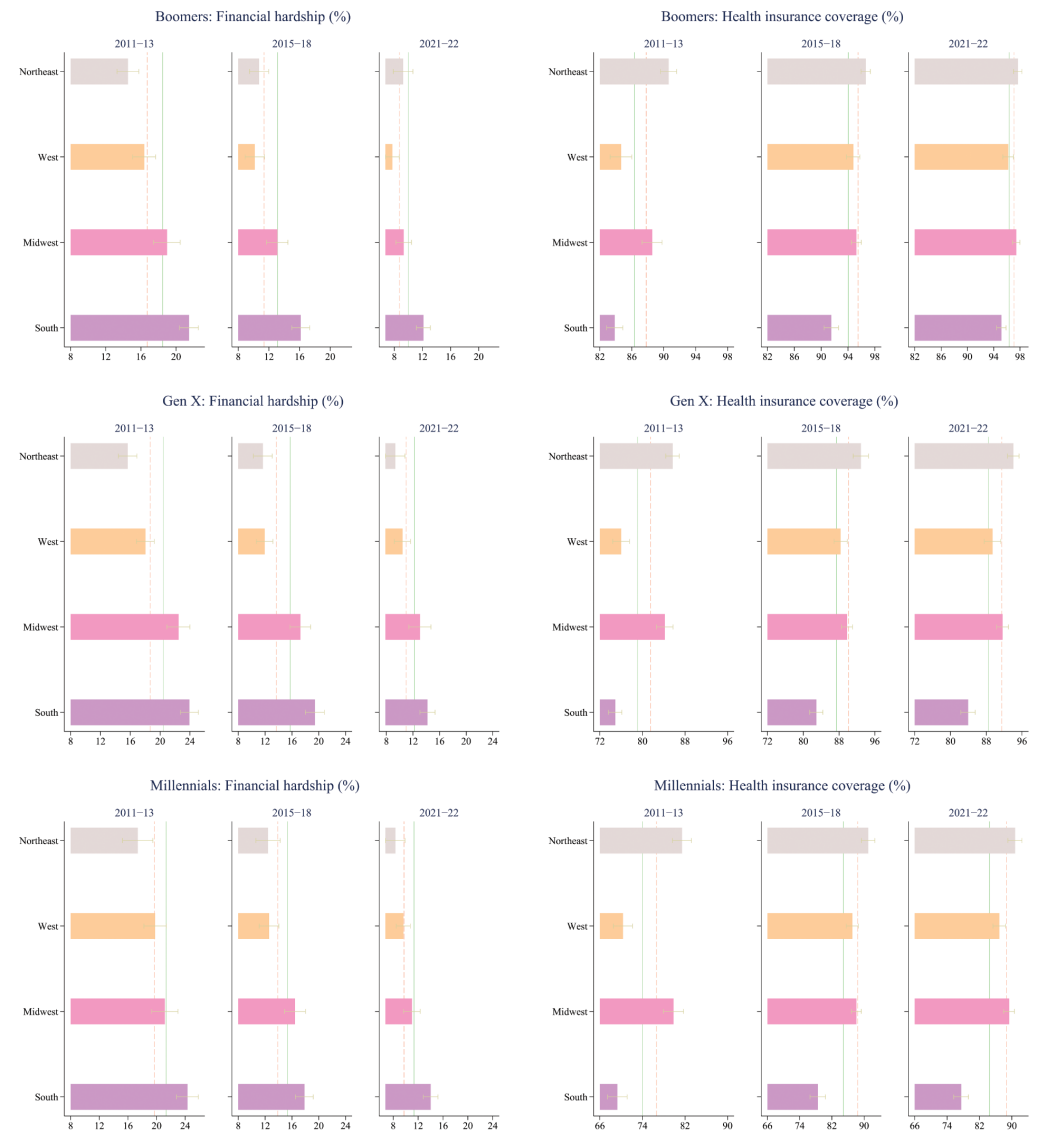
Prevalence of financial hardship and coverage of health insurance among Boomers, Generation X and Millennials across periods. Note: Estimates were obtained using complex survey weights. Horizontal lines across the bars represent 95% confidence intervals. The green solid vertical line shows the national average in respective periods for respective generations. The dashed red vertical line shows national average, excluding South, in respective periods for respective generations

During 2011-13, the proportion of individuals, comprising the three generations, experiencing financial hardship in the US was 20.1%. The proportion declined to 14.7% during 2015-18, and further to 11.2% during 2021-22. Across all periods and for all generations, those who resided in the South had a higher likelihood of experiencing medical financial hardship compared to their respective counterparts residing in the Northeast, Midwest, and West (Fig. 1). For example, during the pre-ACA period, 21.5% of the boomers living in the South experienced financial hardship, which was 6.9 pp (95% CI: 5.3–8.6), 2.5 pp (95% CI: 0.7–4.3) and 5.1 pp (95% CI: 3.4–6.8) higher than that of boomers living in Northeast, Midwest, and West, respectively. During the post-ACA, the share of financial hardship for boomers living in the South declined to 16.2% but was higher by 5.4 pp (95% CI: 3.7–7.1), 3.1 pp (95% CI: 1.2–4.9), and 6.0 pp (95% CI: 4.3–7.7), respectively compared to boomers in other regions. The share further declined to 12.1% during the pandemic and remained higher by 2.9 pp (95% CI: 1.2–4.6), 2.8 pp (95% CI: 1.3–4.3), and 4.4 pp (95% CI: 3.0–5.8) compared to their counterparts living in Northeast, Midwest, and West, respectively. With some exceptions, similar were the cases for generation X and the millennials in respective periods (Fig. 1).

The opposite was the case for health insurance coverage. For all generations across different study periods, those who were living in the South had a lower rate of health insurance coverage compared to their counterparts living in the other three regions. For example, health insurance coverage among generation Xers in the South (74.9%) was the lowest across regions during the pre-ACA period. It increased to 82.9% during post-ACA, and further to 83.9% during the pandemic, but remained the lowest of the four regions in respective periods. Similar were the cases for boomers and millennials in respective study periods (Fig. 1). Thus, boomers, generation Xers, and millennials living in the South had a relatively lower health insurance coverage and relatively higher rate of medical financial hardship, compared to their counterparts living in Northeast, Midwest, and West during all three periods.

The AMEs of residence in the South on financial hardship by pre-ACA, post-ACA, and pandemic periods are presented in Table 2. The likelihood of experiencing financial hardship among boomers, generation Xers, and millennials living in the South was 1.8 to 2.6 pp, 1.8 to 4.0 pp, and 1.7 to 2.8 pp higher, respectively, compared to their counterparts living in rest of the USA.

**Table 2:** Average marginal effects of residence in South on financial hardship by periods

	Without controlling for health insurance			Controlling for health insurance		
	2011–2013	2015–2018	2021–2022	2011–2013	2015–2018	2021–2022
**A. Boomers**						
*residing in South VS. residing in*:						
Rest of U.S.	0.020***	0.026***	0.017**	0.016**	0.022***	0.015**
	(0.009, 0.032)	(0.014, 0.038)	(0.006, 0.028)	(0.005, 0.027)	(0.010, 0.034)	(0.004, 0.027)
Northeast	0.028**	0.026*	0.01	0.022*	0.021	0.007
	(0.008, 0.049)	(0.003, 0.049)	(-0.014, 0.033)	(0.001, 0.042)	(-0.002, 0.044)	(-0.016, 0.031)
Midwest	0.009	0.015	0.012	0.005	0.011	0.01
	(-0.012, 0.029)	(-0.006, 0.035)	(-0.006, 0.03)	(-0.015, 0.025)	(-0.009, 0.032)	(-0.008, 0.029)
West	0.026**	0.038***	0.029***	0.024*	0.034**	0.027**
	(0.006, 0.047)	(0.015, 0.06)	(0.01, 0.047)	(0.004, 0.045)	(0.011, 0.056)	(0.008, 0.046)
Obs.	28,964	26,635	18,895	28,921	26,590	18,862
**B. Gen X**						
*residing in South VS. residing in*:						
Rest of U.S.	0.025***	0.040***	0.018**	0.018**	0.032***	0.013
	(0.012, 0.038)	(0.026, 0.055)	(0.005, 0.032)	(0.005, 0.032)	(0.018, 0.046)	(-0.000, 0.027)
Northeast	0.049***	0.057***	0.028*	0.04***	0.047***	0.024
	(0.027, 0.072)	(0.032, 0.082)	(0.003, 0.054)	(0.018, 0.063)	(0.021, 0.073)	(-0.002, 0.049)
Midwest	0.004	0.019	0.004	-0.003	0.012	0.000
	(-0.02, 0.029)	(-0.006, 0.043)	(-0.021, 0.03)	(-0.028, 0.021)	(-0.012, 0.036)	(-0.025, 0.025)
West	0.029**	0.049***	0.024*	0.026**	0.041***	0.019
	(0.009, 0.05)	(0.026, 0.073)	(0.002, 0.046)	(0.005, 0.047)	(0.017, 0.065)	(-0.004, 0.041)
Obs.	25,366	19,222	13,353	25,315	19,186	13,330
**C. Millennials**						
*residing in South VS. residing in*:						
Rest of U.S.	0.028**	0.017*	0.027***	0.019*	0.007	0.021**
	(0.011, 0.045)	(0.003, 0.032)	(0.014, 0.040)	(0.002, 0.036)	(-0.007, 0.021)	(0.007, 0.034)
Northeast	0.043*	0.024	0.033*	0.029	0.011	0.026
	(0.005, 0.08)	(-0.007, 0.054)	(0.005, 0.06)	(-0.009, 0.067)	(-0.019, 0.041)	(-0.002, 0.054)
Midwest	0.021	-0.001	0.023*	0.011	-0.009	0.017
	(-0.008, 0.05)	(-0.026, 0.025)	(0.001, 0.045)	(-0.017, 0.038)	(-0.034, 0.016)	(-0.005, 0.039)
West	0.026	0.031**	0.027**	0.021	0.020	0.020
	(-0.002, 0.053)	(0.007, 0.056)	(0.006, 0.048)	(-0.005, 0.048)	(-0.004, 0.045)	(-0.001, 0.042)
Obs.	21,057	19,322	13,875	20,965	19,235	13,847

Note: Comparisons of South vs. rest of the U.S. were from Eqs. 3 and 4. Comparison of South vs. other regions were from Eqs. 1 and 2. Models were estimated using complex survey weights. Standard errors for average marginal effects were estimated using the Delta-method. 95% confidence intervals are in parenthesis. Confidence intervals for pair-wise comparisons (e.g., South vs. Northeast) were Bonferroni adjusted. *** *p* < 0.001, ** *p* < 0.01, * *p* < 0.05. Adjustments were made for age, age squared, sex, race and ethnicity, educational attainment, family income (as share of Federal Poverty Line threshold), chronic comorbidity, and survey year fixed effects

Southerner boomers had a higher risk of financial hardship compared to their Western and Northeastern counterparts during both pre- and post- ACA periods. While boomers in the South, compared to boomers in the West, were 2.9 pp more likely to have financial hardship during the pandemic, no statistically significant differences in probabilities were observed between Southerner- and Northeastern- boomers in this period. Differences between Southerner- and Midwestern- boomers were also not statistically significant for any periods.

Generation Xers from the South were more likely to have financial hardship across all three periods compared to their counterparts from Northeast and West. Like the boomers, the differences were not statistically significant South vs. Midwest at any period. Regional differences among millennials, on the other hand, were mixed. While, Southerner millennials, during the pandemic, had 3.3 pp, 2.3 pp, and 2.7 pp higher likelihood of experiencing financial hardship compared to their peers from Northeast, Midwest, and West, respectively, the probabilities were only higher compared to Northeastern millennials in pre-ACA and Western millennials in post-ACA periods.

After accounting for health insurance coverage, the AMEs of residence in the South became relatively smaller or statistically insignificant for all generations across all periods. For example, the higher likelihood of financial hardship during the pandemic among Southerner millennials, compared to their Northeastern, Midwestern, and Western counterparts was no more statistically significant when insurance coverage was adjusted in the model. Given the regional disparities in health insurance coverage, these results suggested that the relationship between residing in the South and experiencing financial hardship might be channeled through the difference in health insurance coverage.

**Fig. 2:**
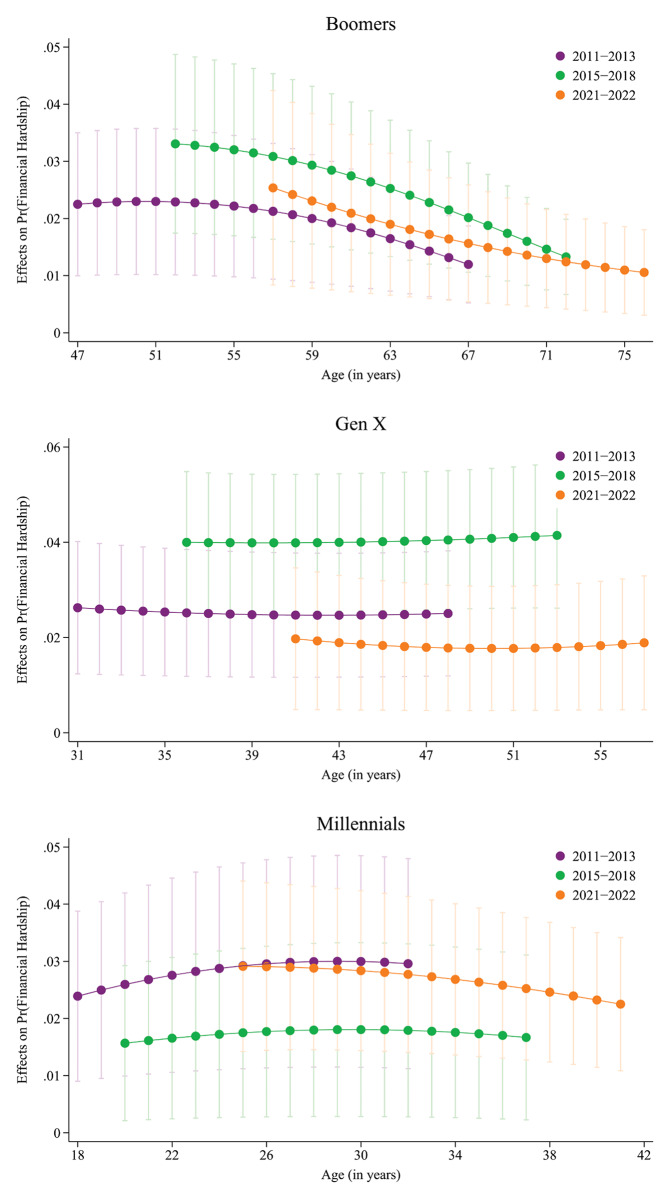
Average marginal effects of residence in South on financial hardship at different ages

Figure 2 presents the AMEs of residence in the South at different ages for the generations across periods. Among boomers, AMEs tend to slightly decrease with age. For example, during the pandemic, Southern boomers at age 60 years were 2.2 pp more likely to have financial hardship compared to their counterparts in rest of the USA, while that difference was 1.4 pp at age 70 years and 1.1 pp at age 75 years. Among generation Xers, the AMEs at different ages were fairly similar within respective periods. Among millennials, a marginally increasing trend during the pre-ACA period and a marginally decreasing trend during the pandemic period were observed.

The sensitivity analyses results by different sociodemographic characteristics are presented in supplemental Tables 1–5. Southerners of all generations, who were female, non-Hispanic Black, had educational attainment of less than a college degree, and had family income of less than 400% of FPL, were more likely to experience financial hardship compared to their respective counterparts in different periods. Southerners of all generations with multiple chronic comorbidities, especially 3 + comorbidities, also had a significantly higher likelihood of having financial hardship in all three periods. In contrast, Southerners of all generations with family income ≥ 400% of FPL and millennials Southerners with a college degree had significantly lower likelihoods of experiencing financial hardship during the pre- and post- ACA periods. Thus, some variations in the relationship between Southern residence and financial hardship were observed at different demographic and socioeconomic levels.

Lastly, results of the KHB decomposition are presented in Table 3. For boomers, 8.6 to 14.0% of the relationship between residing in the South and experiencing medical financial hardship was mediated by health insurance coverage. Among generation Xers, 13.1 to 34.7% of the relationship was mediated by health insurance. Interestingly, for the millennials, 91.1% and 69.1% of the relationship during pre- and post- ACA periods, respectively, were mediated by health insurance coverage. This share, however, came down to 21.1% during the COVID-19 pandemic.

**Table 3:** Karlson, Holm, and Breen (KHB) decomposition results

	2011-13	2015-18	2021-22
**A. Boomers**			
Total effect	0.146***	0.216***	0.150**
	(0.076, 0.216)	(0.143, 0.288)	(0.044, 0.257)
Direct effect	0.126***	0.196***	0.137*
	(0.056, 0.196)	(0.123, 0.269)	(0.031, 0.244)
Indirect effect	0.021***	0.019***	0.013***
	(0.014, 0.027)	(0.014, 0.025)	(0.007, 0.019)
Confounding ratio	1.16	1.10	1.09
Confounding percentage	14.01	8.99	8.56
Observations	28,921	26,590	18,862
**B. Generation X**			
Total effect	0.100**	0.295***	0.206***
	(0.038, 0.163)	(0.208, 0.383)	(0.096, 0.316)
Direct effect	0.066*	0.257***	0.169**
	(0.003, 0.128)	(0.169, 0.345)	(0.056, 0.282)
Indirect effect	0.035***	0.039***	0.037***
	(0.027, 0.042)	(0.029, 0.048)	(0.027, 0.048)
Confounding ratio	1.53	1.15	1.22
Confounding percentage	34.72	13.06	18.06
Observations	25,315	19,186	13,330
**C. Millennials**			
Total effect	0.052	0.080	0.226***
	(-0.016, 0.121)	(-0.002, 0.161)	(0.115, 0.336)
Direct effect	0.005	0.025	0.178**
	(-0.064, 0.074)	(-0.058, 0.107)	(0.067, 0.289)
Indirect effect	0.048***	0.055***	0.048***
	(0.037, 0.059)	(0.044, 0.066)	(0.035, 0.060)
Confounding ratio	11.23	3.23	1.27
Confounding percentage	91.10	69.13	21.11
Observations	20,965	19,235	13,847

Note: 95% confidence intervals are in parenthesis. *** *p* < 0.001, ** *p* < 0.01, * *p* < 0.05. Standard errors were obtained by bootstrapping with 100 replications. Adjustments were made for age, age squared, sex, race and ethnicity, educational attainment, household income (as share of Federal Poverty Line threshold), chronic comorbidity, and survey year fixed effects

## Discussion

The results of this nationally representative, population-based study indicate a persistent regional disparity in experiencing medical financial hardship in the US during the last decade. The frequency of medical financial hardship has declined from the pre-ACA period (2011 to 2013) during the post-ACA (2015 to 2018), and further declined during the COVID-19 pandemic (2021 to 2022) both at the national level and at regional level. However, the difference between South and rest of the U.S. persisted across all three periods. This was evident for individuals representing different generations (i.e., boomers, generation X, and millennials).

These results were robust after adjusting for sociodemographic characteristics along with educational attainment and family income. Furthermore, regional disparities in financial hardship against Southerners were observed for individuals with different demographic and socioeconomic attributes. For example, though compared to White adults in the South, the likelihood of medical financial hardship was slightly higher among Black adults in the South, the regional disparity was evident for both White and Black Southerners of different generations. Black populations are particularly vulnerable to medical financial hardship because they have higher prevalence of comorbidities, have higher out-of-pocket expenses, and have fewer financial resources when compared to their White counterparts [30]. Despite having a larger Black population, compared to that in rest of the US, our results suggested that the higher risk of financial hardship in the South could not be fully explained by differences in racial and ethnic composition.

Similarly, except for college graduate adults and adults with family income of ≥ 400% of FPL, the higher risk of financial hardship was observed in all other educational and income groups, of course in varying degree across generations and periods. These suggest that demographic differences or differences in socioeconomic status conditions of the residents across the regions were not the underlying forces behind the differences in medical financial hardship between the South and rest of the US. However, when we accounted for health insurance coverage, the magnitudes of the relationship declined for all generations across periods, suggesting a partial mediation through the regional differences in availability and access of health insurance. This mediation channel was also confirmed by the results of the KHB decomposition analysis by periods and generations.

The ACA expanded Medicaid eligibility to cover more low-income individuals and families in states that opted to participate in the expansion. States that expanded Medicaid had lower rates of medical financial hardship compared to states that did not expand Medicaid. There is ample evidence suggesting that population health benefits from the expansion of Medicaid. For example, a study by Khatana et al. reported a significant improvement in cardiovascular mortality rates among middle-aged adults associated with Medicaid expansion [31]. With limited Medicaid expansion, Southerners, especially from low-income families, might be deprived of such health improvements and have poorer health outcomes. Evidence suggests that the most prevalent chronic diseases in the US population are highly concentrated in the Southeastern states [32]. Financial distress may occur from chronic multimorbidity [33] and can be exacerbated in absence of the safeguards of affordable health insurance coverage. Goodson et al. observed that Southern residents with two or more chronic conditions were significantly less likely to have insurance coverage compared to their counterparts in the Northeast, West, and Midwest [34]. Our finding that the extent of the relationship between living in the South and experiencing financial hardship was higher among individuals with multiple chronic conditions thus, was in concordance with the findings in extant studies. Of note, Southerners with multimorbidity had higher risk of medical financial hardship compared to their counterparts with multimorbidity in the rest of US.

Research suggests that there are marked disparities in diseases and health risk factors among southern states compared to other states in the US. A systematic analysis across several data sources including the Behavioral Risk Factor Surveillance System (BRFSS), NHIS, National Health and Nutrition Examination Surveys (NHANES), and state inpatient databases found that southern states had higher rates of several health risk factors, including physical inactivity, smoking, and obesity, as well as higher rates of several chronic diseases, including diabetes, stroke, and heart disease [35]. The higher risk of medical financial hardship among Southerners, after accounting of multiple chronic conditions, further suggests that the disparity was not simply due to the regional differences in prevalence rates of disease conditions.

As such, perhaps the disparities may be due to intersectionality of a range of factors, including socioeconomic conditions, political ideology, and psychosocial attributes. Endeavors addressing these disparities will require a multifaceted approach that addresses both individual and structural factors, entailing sustainable and culturally tailored community engaged partnerships. Our findings show the need for future research endeavors, promoting assimilation of multidisciplinary perspectives, to reduce medical financial hardship in the South.

The current analysis has several strengths. First, use of the NHIS data provided high-quality and nationally representative estimate of the regional differences in medical financial hardship across periods. Second, to our knowledge, this is the first study to explore this issue for the boomers, generation Xers, and millennials. These generations had different lived experiences, particularly in the Southern context, and, therefore, were expected to be differentially impacted by national and sub-national policies. The persistent finding across these generations was supportive of the robustness of the relationship. Third, our estimates were adjusted for socioeconomic status conditions (e.g., income and education attainment) that are closely related to financial distress. Further we were able to account for chronic disease conditions, treatment costs of which can also influence financial hardship. However, a few limitations need to be considered. First, financial hardship was a subjective measure reported by individual respondents and was not calculated based on any objective threshold. Second, the NHIS does not report state identifiers, which restrain us controlling for state level attributes. Third, chronic conditions were self-reported and were not confirmed through medical records. Lastly, because of the change in the NHIS sampling frame in 2019, we could not pool data for the entire period (i.e., 2011–2022) to conduct analysis using indicator variables denoting different periods. As such, we were only able to assess disparities within periods and to examine whether disparities persisted across periods. We were not able to assess any changes in disparities across periods.

## Conclusion

In conclusion, we found that boomers, generation Xers, and millennials living in the South were more likely to experience financial hardship compared to their counterparts living in the rest of the US. This relationship was held across different periods, before and after implementation of the ACA and during the COVID-19 pandemic. The relationship was partially mediated via regional differences in health insurance coverage. The results were robust after accounting for critical sociodemographic attributes including age, sex, and race and ethnicity; socioeconomic conditions including education and income; and chronic comorbidities. As such, medical financial hardship may be deemed as an intrinsic public health problem in the South, which could lead to a cycle of health disparities, including limited access to healthcare, increased stress, and higher rates of chronic diseases. Multipronged public health initiatives targeting financial insecurity in the United States’ southern states, therefore, are essential to improve the overall health and well-being of communities in the region.

## Electronic supplementary material

Below is the link to the electronic supplementary material.

Supplementary Material 1
