# Supplementary material for: Medical financial hardship in the Southern United States: the struggle continues across generations pre- and post- the Affordable Care Act
Source: Res Health Serv Reg. 2024 Sep 4;3:13. doi: 10.1007/s43999-024-00049-7 (PMC11371974; doi:10.1007/s43999-024-00049-7)
Supplement: Supplementary file 1 — Supplementary Material 1 [file 43999_2024_49_MOESM1_ESM.docx]

**Supplemental Appendix**

**Supplemental Table 1**. Average marginal effects of residence in South on financial hardship by race and ethnicity

|  | Without controlling for health insurance | | | Controlling for health insurance | | |
| --- | --- | --- | --- | --- | --- | --- |
|  | 2011-2013 | 2014-2018 | 2019-2022 | 2011-2013 | 2014-2018 | 2019-2022 |
|  |  |  |  |  |  |  |
| **A. Boomers** |  |  |  |  |  |  |
| Male | 0.016* | 0.022*** | 0.008 | 0.012 | 0.018** | 0.006 |
|  | (0.002, 0.030) | (0.009, 0.036) | (-0.005, 0.021) | (-0.002, 0.026) | (0.004, 0.031) | (-0.007, 0.019) |
| Female | 0.024*** | 0.028*** | 0.025*** | 0.020** | 0.025*** | 0.023** |
|  | (0.010, 0.037) | (0.014, 0.043) | (0.011, 0.038) | (0.007, 0.033) | (0.010, 0.039) | (0.009, 0.037) |
| **B. Gen X** |  |  |  |  |  |  |
| Male | 0.009 | 0.023** | 0.001 | 0.000 | 0.013 | -0.004 |
|  | (-0.008, 0.025) | (0.006, 0.041) | (-0.015, 0.018) | (-0.016, 0.017) | (-0.004, 0.031) | (-0.021, 0.013) |
| Female | 0.040*** | 0.054*** | 0.033*** | 0.035*** | 0.048*** | 0.029*** |
|  | (0.025, 0.055) | (0.037, 0.072) | (0.016, 0.050) | (0.020, 0.050) | (0.030, 0.065) | (0.012, 0.046) |
| **C. Millennials** |  |  |  |  |  |  |
| Male | 0.014 | 0.009 | 0.010 | 0.002 | -0.005 | 0.001 |
|  | (-0.008, 0.035) | (-0.010, 0.028) | (-0.006, 0.026) | (-0.019, 0.024) | (-0.024, 0.013) | (-0.015, 0.017) |
| Female | 0.042*** | 0.026** | 0.041*** | 0.036*** | 0.020* | 0.038*** |
|  | (0.023, 0.061) | (0.008, 0.043) | (0.024, 0.058) | (0.017, 0.055) | (0.002, 0.037) | (0.022, 0.055) |
|  |  |  |  |  |  |  |

Note: Models were estimated using complex survey weights. Standard errors for average marginal effects were obtained using the Delta-method. 95% confidence intervals are in parenthesis. *** p < 0.001, ** p < 0.01, * p <0.05. Estimates were from the following logistic regressions: $logit\left( {FH}_{i} \right)=\delta_{0}+\sum_{j=1}^{2} \rho_{j}\left( South_{i}\times Sex_{j} \right)+\boldsymbol{X}_{\boldsymbol{i}}\boldsymbol{\delta}_{\boldsymbol{2}}$ and $logit\left( {FH}_{i} \right)=\eta_{0}+\sum_{j=1}^{2} \sigma_{j}\left( South_{i}\times Sex_{j} \right)+\boldsymbol{X}_{\boldsymbol{i}}\boldsymbol{\eta}_{\boldsymbol{2}}+\eta_{3}Insurance_{i}$. The vector ***X*** included age, age squared, race and ethnicity, educational attainment, family income (as share of Federal Poverty Line threshold), chronic comorbidity, and survey year fixed effects.

**Supplemental Table 2**. Average marginal effects of residence in South on financial hardship by race and ethnicity

|  | Without controlling for health insurance | | | Controlling for health insurance | | |
| --- | --- | --- | --- | --- | --- | --- |
|  | 2011-2013 | 2014-2018 | 2019-2022 | 2011-2013 | 2014-2018 | 2019-2022 |
|  |  |  |  |  |  |  |
| **A. Boomers** |  |  |  |  |  |  |
| White | 0.016* | 0.022*** | 0.010 | 0.014* | 0.019** | 0.009 |
|  | (0.002, 0.031) | (0.010, 0.034) | (-0.001, 0.021) | (0.000, 0.028) | (0.007, 0.031) | (-0.002, 0.020) |
| Black | 0.050*** | 0.052*** | 0.031** | 0.048*** | 0.052*** | 0.031** |
|  | (0.032, 0.069) | (0.033, 0.071) | (0.011, 0.050) | (0.030, 0.067) | (0.033, 0.070) | (0.012, 0.050) |
| Asian | -0.021 | 0.024 | 0.020 | -0.026 | 0.022 | 0.018 |
|  | (-0.095, 0.052) | (-0.031, 0.078) | (-0.047, 0.086) | (-0.100, 0.047) | (-0.031, 0.076) | (-0.048, 0.084) |
| Hispanic | 0.031** | 0.026 | 0.048** | 0.019* | 0.015 | 0.042** |
|  | (0.012, 0.050) | (-0.009, 0.060) | (0.018, 0.079) | (0.000, 0.038) | (-0.018, 0.047) | (0.012, 0.072) |
| Other | 0.061 | 0.074 | 0.009 | 0.055 | 0.057 | 0.010 |
|  | (-0.018, 0.140) | (-0.021, 0.168) | (-0.051, 0.069) | (-0.020, 0.130) | (-0.037, 0.152) | (-0.050, 0.070) |
| **B. Gen X** |  |  |  |  |  |  |
| White | 0.035*** | 0.049*** | 0.017* | 0.033*** | 0.044*** | 0.015 |
|  | (0.018, 0.053) | (0.031, 0.067) | (0.002, 0.032) | (0.016, 0.051) | (0.026, 0.062) | (-0.000, 0.030) |
| Black | 0.040*** | 0.054*** | 0.034** | 0.036*** | 0.051*** | 0.033** |
|  | (0.020, 0.059) | (0.030, 0.077) | (0.011, 0.057) | (0.017, 0.054) | (0.028, 0.074) | (0.010, 0.055) |
| Asian | -0.071** | -0.039 | 0.008 | -0.077*** | -0.043 | 0.009 |
|  | (-0.115, -0.027) | (-0.087, 0.010) | (-0.051, 0.067) | (-0.121, -0.033) | (-0.092, 0.006) | (-0.051, 0.069) |
| Hispanic | 0.018 | 0.030* | 0.014 | 0.000 | 0.012 | -0.001 |
|  | (-0.005, 0.042) | (0.001, 0.059) | (-0.009, 0.037) | (-0.024, 0.024) | (-0.017, 0.041) | (-0.023, 0.022) |
| Other | 0.013 | 0.000 | 0.046 | 0.003 | -0.007 | 0.040 |
|  | (-0.081, 0.107) | (-0.090, 0.091) | (-0.017, 0.109) | (-0.091, 0.098) | (-0.101, 0.086) | (-0.023, 0.103) |
| **C. Millennials** |  |  |  |  |  |  |
| White | 0.040*** | 0.009 | 0.039*** | 0.040*** | 0.005 | 0.038*** |
|  | (0.020, 0.061) | (-0.009, 0.027) | (0.022, 0.056) | (0.019, 0.060) | (-0.012, 0.023) | (0.021, 0.054) |
| Black | 0.049*** | 0.055*** | 0.035** | 0.039** | 0.048*** | 0.030* |
|  | (0.021, 0.076) | (0.026, 0.085) | (0.009, 0.062) | (0.014, 0.065) | (0.020, 0.076) | (0.004, 0.056) |
| Asian | -0.048 | -0.008 | -0.016 | -0.051 | -0.009 | -0.019 |
|  | (-0.124, 0.028) | (-0.068, 0.052) | (-0.068, 0.037) | (-0.128, 0.026) | (-0.068, 0.050) | (-0.071, 0.032) |
| Hispanic | 0.011 | 0.041** | 0.004 | -0.014 | 0.014 | -0.011 |
|  | (-0.014, 0.036) | (0.015, 0.067) | (-0.018, 0.025) | (-0.039, 0.011) | (-0.012, 0.040) | (-0.033, 0.011) |
| Other | -0.070 | 0.005 | 0.052 | -0.075 | -0.017 | 0.041 |
|  | (-0.184, 0.044) | (-0.091, 0.101) | (-0.008, 0.111) | (-0.185, 0.036) | (-0.108, 0.073) | (-0.015, 0.097) |
|  |  |  |  |  |  |  |

Note: Models were estimated using complex survey weights. Standard errors for average marginal effects were obtained using the Delta-method. 95% confidence intervals are in parenthesis. *** p < 0.001, ** p < 0.01, * p <0.05. Estimates were from the following logistic regressions: $logit\left( {FH}_{i} \right)=\delta_{0}+\sum_{j=1}^{5} \rho_{j}\left( South_{i}\times Race_{j} \right)+\boldsymbol{X}_{\boldsymbol{i}}\boldsymbol{\delta}_{\boldsymbol{2}}$ and $logit\left( {FH}_{i} \right)=\eta_{0}+\sum_{j=1}^{5} \sigma_{j}\left( South_{i}\times Race_{j} \right)+\boldsymbol{X}_{\boldsymbol{i}}\boldsymbol{\eta}_{\boldsymbol{2}}+\eta_{3}Insurance_{i}$. The vector ***X*** included age, age squared, sex, educational attainment, family income (as share of Federal Poverty Line threshold), chronic comorbidity, and survey year fixed effects.

**Supplemental Table 3**. Average marginal effects of residence in South on financial hardship by education

|  | Without controlling for health insurance | | | Controlling for health insurance | | |
| --- | --- | --- | --- | --- | --- | --- |
|  | 2011-2013 | 2014-2018 | 2019-2022 | 2011-2013 | 2014-2018 | 2019-2022 |
|  |  |  |  |  |  |  |
| **A. Boomers** |  |  |  |  |  |  |
| < HS graduate | 0.045*** | 0.049*** | 0.026* | 0.037*** | 0.039*** | 0.020 |
|  | (0.023, 0.067) | (0.028, 0.070) | (0.003, 0.049) | (0.016, 0.058) | (0.018, 0.059) | (-0.003, 0.043) |
| HS diploma | 0.013 | 0.026** | 0.023** | 0.007 | 0.021* | 0.021** |
|  | (-0.004, 0.029) | (0.008, 0.044) | (0.007, 0.039) | (-0.010, 0.023) | (0.003, 0.039) | (0.005, 0.037) |
| Some college | 0.034*** | 0.037*** | 0.019* | 0.031*** | 0.034*** | 0.018* |
|  | (0.018, 0.050) | (0.020, 0.054) | (0.003, 0.035) | (0.015, 0.047) | (0.017, 0.051) | (0.003, 0.034) |
| College graduate | -0.011 | -0.008 | -0.005 | -0.011 | -0.009 | -0.004 |
|  | (-0.032, 0.009) | (-0.027, 0.010) | (-0.022, 0.013) | (-0.032, 0.010) | (-0.028, 0.009) | (-0.022, 0.013) |
| **B. Gen X** |  |  |  |  |  |  |
| < HS graduate | 0.037** | 0.065*** | 0.025 | 0.017 | 0.041** | 0.009 |
|  | (0.011, 0.063) | (0.032, 0.098) | (-0.006, 0.057) | (-0.007, 0.042) | (0.010, 0.072) | (-0.020, 0.038) |
| HS diploma | 0.024* | 0.040*** | 0.020* | 0.015 | 0.028* | 0.014 |
|  | (0.002, 0.045) | (0.018, 0.062) | (0.001, 0.040) | (-0.007, 0.036) | (0.006, 0.049) | (-0.005, 0.033) |
| Some college | 0.049*** | 0.054*** | 0.027* | 0.044*** | 0.049*** | 0.024* |
|  | (0.030, 0.068) | (0.033, 0.074) | (0.006, 0.048) | (0.025, 0.063) | (0.028, 0.069) | (0.003, 0.045) |
| College graduate | -0.015 | 0.010 | 0.002 | -0.012 | 0.010 | 0.003 |
|  | (-0.037, 0.008) | (-0.010, 0.031) | (-0.018, 0.023) | (-0.035, 0.011) | (-0.011, 0.030) | (-0.018, 0.024) |
| **C. Millennials** |  |  |  |  |  |  |
| < HS graduate | 0.064*** | 0.060** | 0.032 | 0.039* | 0.025 | 0.015 |
|  | (0.031, 0.096) | (0.018, 0.102) | (-0.006, 0.070) | (0.008, 0.070) | (-0.011, 0.062) | (-0.022, 0.051) |
| HS diploma | 0.071*** | 0.037** | 0.053*** | 0.050*** | 0.020 | 0.042*** |
|  | (0.046, 0.097) | (0.012, 0.061) | (0.030, 0.076) | (0.026, 0.074) | (-0.004, 0.043) | (0.019, 0.065) |
| Some college | 0.032** | 0.035** | 0.030** | 0.026* | 0.027* | 0.026** |
|  | (0.008, 0.055) | (0.014, 0.057) | (0.011, 0.049) | (0.004, 0.049) | (0.005, 0.048) | (0.006, 0.045) |
| College graduate | -0.075*** | -0.039*** | -0.003 | -0.065*** | -0.040*** | -0.004 |
|  | (-0.106, -0.044) | (-0.060, -0.018) | (-0.022, 0.015) | (-0.097, -0.033) | (-0.062, -0.019) | (-0.023, 0.015) |
|  |  |  |  |  |  |  |

Note: Models were estimated using complex survey weights. Standard errors for average marginal effects were obtained using the Delta-method. 95% confidence intervals are in parenthesis. *** p < 0.001, ** p < 0.01, * p <0.05. Estimates were from the following logistic regressions: $logit\left( {FH}_{i} \right)=\delta_{0}+\sum_{j=1}^{4} \rho_{j}\left( South_{i}\times Education_{j} \right)+\boldsymbol{X}_{\boldsymbol{i}}\boldsymbol{\delta}_{\boldsymbol{2}}$ and $logit\left( {FH}_{i} \right)=\eta_{0}+\sum_{j=1}^{4} \sigma_{j}\left( South_{i}\times Education_{j} \right)+\boldsymbol{X}_{\boldsymbol{i}}\boldsymbol{\eta}_{\boldsymbol{2}}+\eta_{3}Insurance_{i}$. The vector ***X*** included age, age squared, sex, race and ethnicity, family income (as share of Federal Poverty Line threshold), chronic comorbidity, and survey year fixed effects.

**Supplemental Table 4**. Average marginal effects of residence in South on financial hardship by income

|  | Without controlling for health insurance | | | Controlling for health insurance | | |
| --- | --- | --- | --- | --- | --- | --- |
|  | 2011-2013 | 2014-2018 | 2019-2022 | 2011-2013 | 2014-2018 | 2019-2022 |
|  |  |  |  |  |  |  |
| **A. Boomers** |  |  |  |  |  |  |
| < 100% FPL | 0.126*** | 0.100*** | 0.072*** | 0.086*** | 0.081*** | 0.065*** |
|  | (0.104, 0.148) | (0.075, 0.126) | (0.042, 0.102) | (0.066, 0.107) | (0.057, 0.105) | (0.036, 0.095) |
| 100 to < 200% FPL | 0.156*** | 0.125*** | 0.079*** | 0.125*** | 0.112*** | 0.074*** |
|  | (0.132, 0.180) | (0.100, 0.150) | (0.055, 0.103) | (0.102, 0.149) | (0.087, 0.138) | (0.050, 0.097) |
| 200 to < 400% FPL | 0.051*** | 0.050*** | 0.027** | 0.046*** | 0.046*** | 0.024** |
|  | (0.033, 0.069) | (0.032, 0.069) | (0.011, 0.043) | (0.028, 0.063) | (0.028, 0.064) | (0.008, 0.040) |
| ≥ 400% FPL | -0.092*** | -0.055*** | -0.042*** | -0.085*** | -0.054*** | -0.041*** |
|  | (-0.109, -0.076) | (-0.070, -0.040) | (-0.055, -0.028) | (-0.102, -0.068) | (-0.070, -0.039) | (-0.055, -0.027) |
| **B. Gen X** |  |  |  |  |  |  |
| < 100% FPL | 0.107*** | 0.090*** | 0.046** | 0.069*** | 0.059*** | 0.032 |
|  | (0.082, 0.131) | (0.056, 0.124) | (0.013, 0.079) | (0.046, 0.093) | (0.027, 0.090) | (-0.000, 0.063) |
| 100 to < 200% FPL | 0.129*** | 0.136*** | 0.076*** | 0.099*** | 0.112*** | 0.062*** |
|  | (0.105, 0.153) | (0.105, 0.167) | (0.046, 0.106) | (0.074, 0.123) | (0.081, 0.143) | (0.033, 0.091) |
| 200 to < 400% FPL | 0.027* | 0.075*** | 0.051*** | 0.023* | 0.067*** | 0.044*** |
|  | (0.006, 0.048) | (0.052, 0.098) | (0.027, 0.074) | (0.002, 0.044) | (0.044, 0.090) | (0.021, 0.068) |
| ≥ 400% FPL | -0.084*** | -0.045*** | -0.037*** | -0.074*** | -0.045*** | -0.037*** |
|  | (-0.101, -0.066) | (-0.062, -0.028) | (-0.053, -0.020) | (-0.092, -0.056) | (-0.062, -0.028) | (-0.054, -0.020) |
| **C. Millennials** |  |  |  |  |  |  |
| < 100% FPL | 0.057*** | 0.029 | 0.060*** | 0.033* | 0.007 | 0.047** |
|  | (0.029, 0.085) | (-0.002, 0.060) | (0.028, 0.092) | (0.007, 0.059) | (-0.022, 0.035) | (0.015, 0.078) |
| 100 to < 200% FPL | 0.081*** | 0.086*** | 0.052*** | 0.058*** | 0.059*** | 0.037** |
|  | (0.053, 0.108) | (0.060, 0.111) | (0.028, 0.075) | (0.032, 0.085) | (0.034, 0.084) | (0.014, 0.061) |
| 200 to < 400% FPL | 0.044** | 0.034** | 0.043*** | 0.037** | 0.027* | 0.037*** |
|  | (0.018, 0.070) | (0.012, 0.055) | (0.022, 0.064) | (0.011, 0.063) | (0.006, 0.049) | (0.017, 0.058) |
| ≥ 400% FPL | -0.088*** | -0.061*** | -0.017 | -0.075*** | -0.060*** | -0.018 |
|  | (-0.118, -0.057) | (-0.082, -0.039) | (-0.036, 0.001) | (-0.107, -0.043) | (-0.082, -0.039) | (-0.037, 0.000) |
|  |  |  |  |  |  |  |

Note: Models were estimated using complex survey weights. Standard errors for average marginal effects were obtained using the Delta-method. 95% confidence intervals are in parenthesis. *** p < 0.001, ** p < 0.01, * p <0.05. Estimates were from the following logistic regressions: $logit\left( {FH}_{i} \right)=\delta_{0}+\sum_{j=1}^{4} \rho_{j}\left( South_{i}\times Income_{j} \right)+\boldsymbol{X}_{\boldsymbol{i}}\boldsymbol{\delta}_{\boldsymbol{2}}$ and $logit\left( {FH}_{i} \right)=\eta_{0}+\sum_{j=1}^{4} \sigma_{j}\left( South_{i}\times Income_{j} \right)+\boldsymbol{X}_{\boldsymbol{i}}\boldsymbol{\eta}_{\boldsymbol{2}}+\eta_{3}Insurance_{i}$. The vector ***X*** included age, age squared, sex, race and ethnicity, education, chronic comorbidity, and survey year fixed effects.

**Supplemental Table 5**. Average marginal effects of residence in South on financial hardship by comorbidity

|  | Without controlling for health insurance | | | Controlling for health insurance | | |
| --- | --- | --- | --- | --- | --- | --- |
|  | 2011-2013 | 2014-2018 | 2019-2022 | 2011-2013 | 2014-2018 | 2019-2022 |
|  |  |  |  |  |  |  |
| **A. Boomers** |  |  |  |  |  |  |
| None | -0.038*** | -0.040*** | -0.026** | -0.046*** | -0.048*** | -0.031*** |
|  | (-0.056, -0.020) | (-0.058, -0.022) | (-0.045, -0.008) | (-0.063, -0.028) | (-0.065, -0.030) | (-0.050, -0.013) |
| 1 | 0.007 | 0.004 | -0.003 | 0.001 | -0.001 | -0.004 |
|  | (-0.009, 0.023) | (-0.014, 0.022) | (-0.020, 0.015) | (-0.015, 0.016) | (-0.018, 0.017) | (-0.021, 0.013) |
| 2 | 0.035*** | 0.032*** | 0.008 | 0.034*** | 0.030** | 0.007 |
|  | (0.015, 0.055) | (0.014, 0.050) | (-0.008, 0.023) | (0.015, 0.054) | (0.012, 0.049) | (-0.009, 0.022) |
| 3+ | 0.123*** | 0.096*** | 0.077*** | 0.127*** | 0.096*** | 0.079*** |
|  | (0.101, 0.145) | (0.077, 0.116) | (0.059, 0.096) | (0.105, 0.149) | (0.077, 0.116) | (0.060, 0.098) |
| **B. Gen X** |  |  |  |  |  |  |
| None | -0.017* | -0.004 | -0.016 | -0.025** | -0.013 | -0.022* |
|  | (-0.032, -0.002) | (-0.023, 0.015) | (-0.033, 0.002) | (-0.041, -0.010) | (-0.031, 0.006) | (-0.039, -0.004) |
| 1 | 0.049*** | 0.054*** | 0.020 | 0.043*** | 0.047*** | 0.017 |
|  | (0.028, 0.070) | (0.034, 0.075) | (-0.001, 0.041) | (0.022, 0.063) | (0.027, 0.068) | (-0.004, 0.037) |
| 2 | 0.157*** | 0.098*** | 0.062*** | 0.157*** | 0.095*** | 0.060*** |
|  | (0.126, 0.187) | (0.071, 0.126) | (0.034, 0.090) | (0.127, 0.188) | (0.068, 0.122) | (0.032, 0.089) |
| 3+ | 0.145*** | 0.147*** | 0.104*** | 0.151*** | 0.146*** | 0.107*** |
|  | (0.103, 0.187) | (0.110, 0.184) | (0.068, 0.139) | (0.110, 0.193) | (0.110, 0.183) | (0.072, 0.143) |
| **C. Millennials** |  |  |  |  |  |  |
| None | -0.001 | -0.003 | 0.006 | -0.012 | -0.015 | -0.000 |
|  | (-0.020, 0.017) | (-0.020, 0.014) | (-0.009, 0.021) | (-0.030, 0.006) | (-0.031, 0.001) | (-0.015, 0.015) |
| 1 | 0.084*** | 0.044*** | 0.050*** | 0.078*** | 0.038*** | 0.044*** |
|  | (0.055, 0.114) | (0.023, 0.065) | (0.028, 0.072) | (0.049, 0.108) | (0.017, 0.059) | (0.022, 0.066) |
| 2 | 0.172*** | 0.098*** | 0.079*** | 0.175*** | 0.090*** | 0.079*** |
|  | (0.112, 0.232) | (0.048, 0.148) | (0.037, 0.121) | (0.118, 0.233) | (0.042, 0.137) | (0.036, 0.122) |
| 3+ | 0.209*** | 0.126*** | 0.178*** | 0.211*** | 0.126*** | 0.181*** |
|  | (0.118, 0.300) | (0.052, 0.201) | (0.094, 0.262) | (0.120, 0.303) | (0.055, 0.198) | (0.099, 0.264) |
|  |  |  |  |  |  |  |

Note: Models were estimated using complex survey weights. Standard errors for average marginal effects were obtained using the Delta-method. 95% confidence intervals are in parenthesis. *** p < 0.001, ** p < 0.01, * p <0.05. Estimates were from the following logistic regressions: $logit\left( {FH}_{i} \right)=\delta_{0}+\sum_{j=1}^{4} \rho_{j}\left( South_{i}\times Comorbidity_{j} \right)+\boldsymbol{X}_{\boldsymbol{i}}\boldsymbol{\delta}_{\boldsymbol{2}}$ and $logit\left( {FH}_{i} \right)=\eta_{0}+\sum_{j=1}^{4} \sigma_{j}\left( South_{i}\times Comorbidity_{j} \right)+\boldsymbol{X}_{\boldsymbol{i}}\boldsymbol{\eta}_{\boldsymbol{2}}+\eta_{3}Insurance_{i}$. The vector ***X*** included age, age squared, sex, race and ethnicity, education, family income (as share of Federal Poverty Line threshold), and survey year fixed effects.
